# Conservation in Prostatic Hypertrophy1Summary of papers read before the Cattaraugus County and Niagara County Medical Societies.

**Published:** 1910-10

**Authors:** Nelson W. Wilson

**Affiliations:** Lecturer on Genitourinary and Venereal Diseases, Medical Department, University of Buffalo; Assistant in Genitourinary Surgery, Buffalo General Hospital; 49 Niagara Street


### Conservation in Prostatic Hypertrophy[1]

By NELSON W. WILSON, M.D.
Lecturer on Genitourinary and Venereal Diseases, Medical Department, University of Buffalo; Assistant in Genitourinary Surgery, Buffalo General Hospital.

THE search for some method of dealing with the hypertrophied prostate in those cases which because of heart conditions or kidney lesions made operation impossible or inadvisable, by which the patient might be given the greatest amount of comfort with the greatest safety to himself, led to a more painstaking consideration of the underlying factors in the hypertrophy and its distressing symptoms. The problem was by no means a new one; the prominent features were the same, the frequency and the urgency of urination, the constant restless, harrowing irritation of the urinary tract with the consequent loss

---

1. Summary of papers read before the Cattaraugus County and Niagara County Medical Societies.

of rest and the ultimate undermining of health, were all too apparent in the majority of cases which came under my care; and earlier efforts at relief were confined merely to cleansing the bladder by irrigations with weak permanganate of potash carefully administered and later a very mild dilatation of the urethra with steel sounds; latterly the steel instruments were replaced by the Eynard bougie. This gave in some cases a moderate amount of relief but it was not sufficient for the results sought. I had already realised the fact so frequently noted that there were cases of prostatic hypertrophy which gave little or no trouble where the organ was of considerable size; and that there were other cases where with a relatively small prostate the disturbance was out of all proportion to the size of the organ. It did not appear that the difference in type whether it were a hard or a soft hypertrophy so called made much difference; the hard as is well known being a true hypertrophy of muscular and connective tissue elements and the soft type being a hyperplasia of the glandular elements; neither did it appear that the arteriosclerosis so frequently found in connection with prostatism had any effect on the condition itself because even in those cases where there was an apparent sclerosis of the vessels of the bladder, the contactile power of the bladder remained good even with marked retention. The comfort following irrigation and dilatation was marked in the majority of cases; so too was the recurrence of the trouble marked and discouraging and the development of what I have boldly termed a new phase of prostatic hypertrophy and the conservative treatment of the condition was purely accidental and rather startling.

I sought a tenable reason for the disturbances accompanying a small prostate whose size was too insignificant to be at the bottom of the urgency, frequency and tenesmus and the case which developed this so-called new phase was of the puzzling character. The patient was 72 years old; his prostate was not one of the bulging type which blocked either the urethra or rectum and the irrigation and dilatation gave him immediate relief. His physical condition contraindicated any other operative procedures. After the treatment he developed an epididymitis which was followed by a discharge of pus from the urethra; pus which was yellow and thick and which was accompanied by moderate burning on urination. He denied any previous venereal history. The pus was examined and found to contain many organisms in which the colon bacillus predominated to such an extent as to practically occupy the field to the exclusion of all others. Up to this time the prostatic urethra had been considered only as an incidental feature in prostatic hypertrophy. An endoscopic examination was made in this case and that portion of urethra was found infected, hemorrhagic to the extent that it bled rather easily on manipulation, and there were numerous patches; from the ejaculatory ducts and the prostatic ducts secretion was obtained which showed the presence of the colon bacillus in relatively large numbers. Irrigations were continued, the prostatic urethra was treated endoscopically and finally by installations and the improvement was so marked as to be startling. This was the beginning of the development of the serious consideration of the colon bacillus as the chief factor in the serious disturbances which accompany prostatic hypertrophy.

The fact that 34 per cent. of cases of prostatic hypertrophy occur after 60 years and that of these 50 per cent. produce symptoms was also an indication that there was something else beside the mere enlargement of the organ to account for the disturbances which follow. On these lines then the field broadened and efforts were bent toward determining the colon infection. I shall not go into the history of this organism other than to mention that when it enters the blood current it is eliminated by the kidneys and is frequently found in the urine; so frequently that it has become to be considered one of the important factors of urinary infections, and that it appears in organs remote from the colon in diseased conditions having been found in the liver, bile kidneys and heart. In the normal bladder it does little damage but in the presence of obstruction its activities are increased and the damage it does is almost incalculable. This has been demonstrated by the introduction of the organism into the circulation of dogs, its appearance in the urine and its subsequent disappearance without treatment; but when introduced into the bladder and there is obstruction to the urinary flow by ligation of the urethra there follows a serious cystitis. I have found in dogs the same condition of prostatic hypertrophy with attendant colon infection that is found in the human. The dog is the only animal having prostatic hypertrophy and infectious urethritis; and as a matter of interest it may be stated that the operation of suprapubic prostatectomy was done on ten dogs before it was performed on man.

These considerations lead me to make this almost revolutionary statement: that hypertrophy of the prostate—by that I mean the physiologic enlargement of the organ—in itself does little damage so long as there is no active infection, irrespective of the claim that all prostatic hypertrophy is due to inflammatory changes; but that when the enlarged prostate is become infected by the colon bacillus, that moment the basework is laid for all the damaging and disastrous conditions which we are called upon to combat and which so frequently end in the death of the patient. This infection does not begin as one might imagine with the startling pictures which most infections elsewhere present. It is a slow process and unfortunately we seldom see these cases until it has become so pronounced that it produces serious symptoms such as tenesmus, with its train of distressing and dangerous consequences.

One of the first indications noted in beginning trouble is the complaint of loss of appetite and digestive disturbances due to diminished kidney power, renal insufficiency and urinary intoxication. This is followed by hyperemia and engorgement of the prostate and infection by the colon bacillus. It is at or near this point that I think we have been overlooking serious symptoms, the first indication of kidney involvement indicated by the relatively large amounts of urine with low specific gravity. This is the first indication of the presence of the urinary vicious circle, ultimately producing serious bladder changes; the increased work causing in many cases a true hypertrophy of the walls of the bladder; there may also be atony and dilatation with fibrous degeneration. The changes on the walls of the bladder are no different than are wrought by cystitis from any cause whatever; there is a vesical catarrh, the ropy mucus of which adds to the obstruction of the urethra; there is congestion and ulcerations and deposits of urinary salts; and where there is large retention sudden withdrawal of the fluid will be followed by hemorrhage of the venous congestion.

Aside from the serious inroads on the patient's health the residual urine undergoes ammoniac decomposition, for while the colon bacillus as a rule only mildly has the power to decompose urea and so give an alkaline urine by itself, in connection with other organisms this condition is produced and the natural consequence is the formation of calculi. Frequently there is epididymitis and even orchitis with prostatic hypertrophy and there is in a majority of cases a seminal vesiculitis of colon bacillus origin. Careful diagnosis should be made as between atrophy of the bladder and prostatic hypertrophy, and the difference between dribbling due to atrophy and retention should be borne in mind; in retention there is residual urine; in atrophy the urethra is shorter, and there is no resistance to sound.

It should be remembered that as a rule in such cases the upper walls of the deep urethra are covered with greatly distended vessels and that careless use of instruments is likely to be followed by serious hemorrhage. Collargolum is used exclusively for the treatment of the deep urethra. Silver nitrate is contraindicated because it has no effect on the infecting organism, irritates the urethra and causes thickening of tissues if used for any length of time.

When it is determined that the colon bacillus is present an autogènous vaccine is prepared and injections given according to the severity of the infection. It has been the custom to await reaction before repeating the dose of vaccine which varies to meet individual conditions. In all those cases where there was not actual mechanic obstruction owing to the size of the prostate itself there was marked relief and several cases were so definitely benefitted as to verge close to a condition resembling actual cure. These latter have not been under observation a sufficient length of time to warrant a definite statement; but the periods between urination continue with slight variation to be comfortably long. One illustrative case will suffice as a preliminary report. The patient was in the prostatic age and was urinating with distressing frequency day and night when admitted to the hospital. With urethral and bladder cleanliness and the conservative and careful use of autogenous colon vaccine he improved to such an extent that when he left the hospital five hours elapsed between urination.

As to the dose of vaccines there is no fixed rule. Each case must be treated on its merits and according to its demands. Even in those cases where there is true hypertrophy of the prostate and actual mechanic obstruction there is possible some measure of relief by the use of vaccines although here prostatectomy is indicated. Where prostatectomy has been done, the discomforts of the operation appear to have been lessened by the use of the vaccine before and after operation.

Since the papers summarised were read, use has been made of the mixed colon vaccines now in the experimental stage, prepared by Parke, Davis & Co., and excellent results have been obtained. At the present time it would seem that this mixed vaccine would replace the autogenous vaccine which has been used.

The results obtained in the series of cases on which these statements are based would indicate that the operation of prostatectomy is wholly unnecessary for the relief of the urinary symptoms in prostatiques until it has been definitely decided that the colon infection is not alone the cause of the trouble.

49 Niagara Street.

---

**Two Cases of Actinomycosis**

Howard Jones, Circleville, Ohio, reports two cases of actinomycosis, in each of which a swelling supposed to be a tooth abscess appeared in the cheek. A characteristic tumor later developed in which the ray fungus was found. One patient was cured by excision, the other had a secondary tumor which was not operated upon.—*Medical Record,* August 27, 1910.

